# Dark trions and biexcitons in WS_2_ and WSe_2_ made bright by e-e scattering

**DOI:** 10.1038/srep45998

**Published:** 2017-04-06

**Authors:** Mark Danovich, Viktor Zólyomi, Vladimir I. Fal’ko

**Affiliations:** 1 National Graphene Institute, University of Manchester, Booth St E, Manchester M13 9PL, UK

## Abstract

The direct band gap character and large spin-orbit splitting of the valence band edges (at the K and K’ valleys) in monolayer transition metal dichalcogenides have put these two-dimensional materials under the spot-light of intense experimental and theoretical studies. In particular, for Tungsten dichalcogenides it has been found that the sign of spin splitting of conduction band edges makes ground state excitons radiatively inactive (dark) due to spin and momentum mismatch between the constituent electron and hole. One might similarly assume that the ground states of charged excitons and biexcitons in these monolayers are also dark. Here, we show that the intervalley (*K* ⇆ *K*′) electron-electron scattering mixes bright and dark states of these complexes, and estimate the radiative lifetimes in the ground states of these “semi-dark” trions and biexcitons to be ~10 p*s*, and analyse how these complexes appear in the temperature-dependent photoluminescence spectra of WS_2_ and WSe_2_ monolayers.

The truly 2D nature of TMDCs^1^,^2^,^3^,^4^,^5^,^6^,^7^ enhances the effects of Coulomb interaction^8^,^9^, resulting in charge complexes such as excitons^10^,^11^,^12^,^13^, trions^13^ and biexcitons^14^ with binding energies that are orders of magnitude larger compared to conventional semiconductors such as GaAs. These complexes, which dominate the optical response of these materials, are comprised of spin/valley polarised electrons and holes residing at the corners K and K′ of the hexagonal Brillouin zone (BZ), where the selection rules of optical transitions require the same spin and valley states of the involved electrons at the conduction and valence band edges. As a result, the opposite spin projections of the conduction (*c*) and valence (*v*) band edges, found in monolayers of WS_2_ and WSe_2_, makes ground state excitons in these 2D crystals dark^15^,^16^, so that their radiative transition would require help from defects, phonons^17^ or magnetic field^18^,^19^.

Applying the spin and valley selection rules to ground state trions and biexcitons might imply that these charge complexes are dark, too. In the ‘dark’ (*d*) state both electrons are in the bottom spin-orbit split states of *c*-band, whereas in the state to be ‘bright’ (*b*), one of the electrons has to be in the excited spin-split state. Here, we show that an intervalley scattering^20^,^21^ of the *c*-band electrons mixes dark and bright states of complexes (Fig. 1), hence transferring some optical strength from *b*- to *d*-states and making dark state ‘semi-dark’. For the resulting recombination line of such semi-dark complexes, we find that it is shifted downwards in energy (relative to the bright trion line) by 2Δ_*SO*_, twice the *c*-band spin-orbit splitting.

With the reference to Fig. 1, the basis of trion, T (biexciton, B) states, 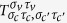 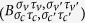, can be described by spin, *σ* = ↑, ↓ and valley, *τ* = *K, K*′ quantum numbers of their constituent *c*- and *v*-band states. In these notations, dark ground state exciton complexes *T*_*d*_ (*B*_*d*_) are 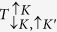 and 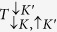 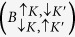, and the excited states 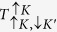 and 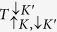 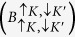 are bright, *T*_*b*_ (*B*_*b*_) (Supplementary material S1). These states are mixed by the intervalley interaction illustrated by a sketch in Fig. 1


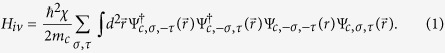


Here, 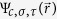 are the conduction band electron field operators. The large momentum transfer between two electrons changing their valley states is determined by their Coulomb interaction at the unit cell scale, parametrised by a dimensionless factor 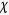. We estimate the size of this factor using both a tight-binding model and density functional theory (DFT). For the tight-binding model, we use the DFT calculated orbital decomposition to construct the Bloch states at the Brillouin zone corners, and we use a 3D Coulomb potential for the interaction between electrons. As the *c*-band states at the *K/K*′ points are primarily composed^6^,^7^ of the metal 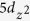 orbitals centred at the lattice sites 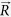 of metallic atoms in TMDC lattice, 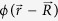, which we use to construct the tight-binding model Bloch states, to find


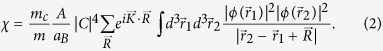


Here, 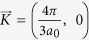 with *a*_0_ the lattice constant of WX_2_, 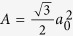 is the unit cell area, *m*_*c*_ is the *c*-band electron effective mass, *m* is the free electron mass, *a*_*B*_ is the Bohr radius, and *C* is the transition metal 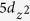 orbital amplitude in the *c*-band edge at the *K* point (supplementary material S2.2). Similarly, we evalutaed 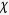 from wave functions obtained using DFT implemented in the local density approximation and VASP^22^ code (neglecting spin-orbit coupling). We used a plane-wave basis corresponding to 600 eV cutoff energy and a 12 × 12 grid of k-points in the 2D Brillouin zone. We also had to employ periodic boundary conditions in the *z*-direction; for this reason we used a large inter-layer distance of 20 Å to mimic the limit of an isolated monolayer. The form factor was calculated by post-processing the DFT wave functions, by taking the matrix element of the bare Coulomb interaction between the initial and final states of the scattering process (see supplementary material S2.1). These two calculations have returned values of the intervalley scattering factor 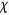, as listed in Table 1. In the basis of 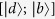 of dark and bright states of trions, 
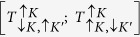
 and 
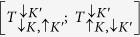
, or biexcitons 
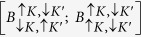
, the coupling in equation (1) leads to the mixing described by a 2 × 2 matrix


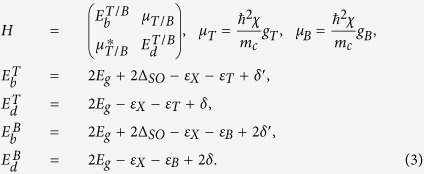


Where *E*_*g*_ is the band gap, 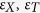, and 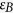 are the exciton, trion, and biexciton binding energies, respectively, and 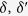 stand for the intravalley and intervalley electron-hole exchange^23^, *δ* ≈ 6 meV, which we will neglect in the following calculations. Note that the effective masses of the *c*-band spin split bands differ by^7^ ~30–40% with the lower bands having the higher effective electron mass. This results in slightly higher binding energies for the dark ground state charge complexes compared to the excited states, resulting in a larger value for their energy difference *E*_*b*_ − *E*_*d*_. The mixing parameter 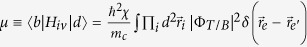, (where 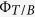 stands for the wave function of the trion or biexciton and 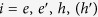, is determined by the electron-electron contact pair densities^24^ in the trion, *g*_*T*_ and biexciton, *g*_*B*_. The mixing of the dark and bright states results in a slight shift of their energies and, most importantly, in a finite radiative decay rate, 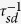 of the semi-dark (*sd*) trions (*T*) and biexcitons (*B*),


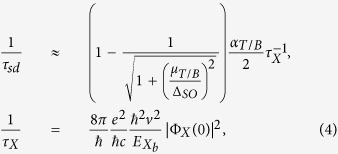


where 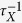 is the radiative decay rate of the bright exciton^25^,^26^,^27^, determined by the electron-hole overlap factor 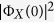 (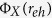 is the envelope wave function describing relative motion of the electron and hole in the exciton), *v* is the velocity related to the off diagonal momentum matrix element. The values of the factors 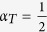 and *α*_*B*_ = 1 have been estimated based on the following consideration (see supplementary material S3). As the exciton’s binding energy is significantly larger than that of the trion or biexciton, these bound complexes can be viewed as strongly-bound, with an additional weakly bound electron in the case of a trion, or an exciton in the case of a biexciton. For a trion, this results in a reduction of the recombining electron-hole contact pair density by a factor of two as compared to the exciton, as the hole is shared between the two electrons such that the recombining electron (which has the right spin projection), will be near it only half of the time. In the case of the biexciton, each electron spends half of the time near its hole with which it can recombine, and half of the time near the other hole. As there are two excitons able to recombine we get *α*_*B*_ = 1. The resulting values for the lifetimes (using the material parameters in Table 2) are summarized in Table 1.

The mixing of the dark and bright states produces photoluminescence lines shown schematically in Fig. 2. The emitted photon energies of these lines are determined by both the binding energies and the shake-up into the higher-energy spin-split *c*-band in the final state,


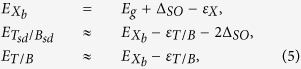


Being the ground states, the semi-dark trion and biexcitons (*T*_*sd*_, *B*_*sd*_) do not require an activation and therefore should appear in the spectrum even at low temperatures. In contrast, the bright states do require thermal activation, resulting in a 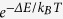 temperature dependence of their lines intensities. For the bright exciton, trion 
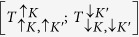
 and biexciton 
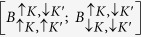
 we have Δ*E* ≈ Δ_*SO*_, while for the excited mixed dark and bright trion (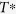) 
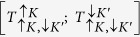
 and biexciton (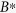) 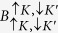, 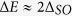. Also, the presence of a final state electron or exciton results in an antisymmetric line shape with a cutoff due to the recoil kinetic energy of the remaining electron or exciton that shifts the emission line to a lower energy. A typical recoil kinetic energy is 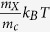 for the trions and *k*_*B*_*T* for biexcitons, with *k*_*B*_ the Boltzmann constant, *m*_*X*_ the exciton mass, and *m*_*c*_ the *c*-band electron effective mass.

In conclusion, we have shown that intervalley electron-electron scattering makes “dark” ground state trions and biexcitons in Tungsten dichalcogenides WS_2_ and WSe_2_ optically active, with a lifetime *τ*_*T/B*_ ~ 10 p*s*, to compare with a sub-ps lifetime of bright excitons in 2D TMDCs.

## Additional Information

**How to cite this article:** Danovich, M. *et al*. Dark trions and biexcitons in WS_2_ and WSe_2_ made bright by e-e scattering. *Sci. Rep.* **7**, 45998; doi: 10.1038/srep45998 (2017).

**Publisher's note:** Springer Nature remains neutral with regard to jurisdictional claims in published maps and institutional affiliations.

## Supplementary Material

Supplementary Materials

## Figures and Tables

**Figure 1:**
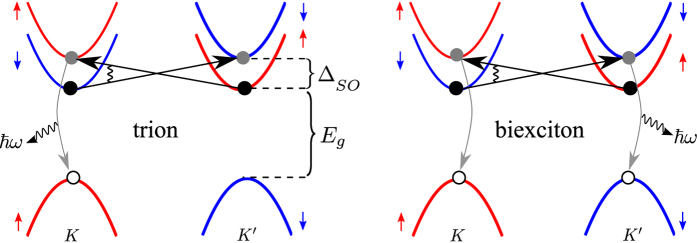
Intervalley electron-electron scattering process. Schematics of the band structures of W*X*_2_ near the *K, K*′ points of the BZ, and the intervalley scattering process that mixes dark and bright states of trions (T) and biexcitons (B). *E*_*g*_ is the band gap and Δ_*SO*_ stands for the conduction band spin splitting. Due to the large spin-orbit splitting in the valence band, the valence band is shown only for the higher-energy spin-polarised states.

**Figure 2:**
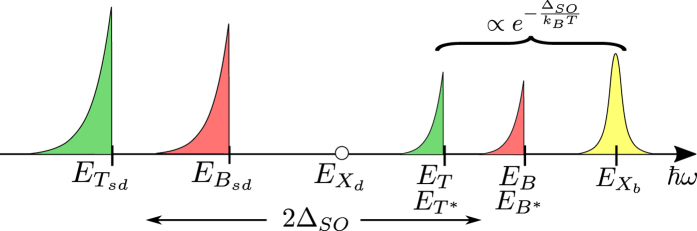
Low temperature photoluminescence spectrum of WX_2_. Sketch of the low temperature (*k*_*B*_*T* < Δ_*SO*_) photoluminescence spectrum of WX_2_ including the bright exciton, dark and bright trions (green) and dark and bright biexcitons (red). The excited bright trions and excitons are denoted by T^*^ and B^*^. The dark exciton (*X*_*d*_) energy is marked as a reference point 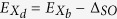.

**Table 1:** Scattering matrix elements and radiative lifetimes.

	*χ*_*DFT*_	*χ*_*TB*_	*μ*_*T*_	*μ*_*B*_	*τ*_*X*_	*τ*_*sd*_*(T)*	*τ*_*sd*_*(B)*
			[meV]	[meV]	[ps]	[ps]	[ps]
WS_2_	1.0	1.6	18[29]	8.6[13]	0.25	7.8[3.9]	15[7.0]
WSe_2_	1.3	2.0	19[30]	9.2[14]	0.26	9.4[4.7]	18[8.0]

Listed are the Intervalley scattering parameter *χ* calculated using DFT and tight binding (TB) model and the corresponding trion and biexciton mixing parameters *μT/B* obtained using the electron-electron contact pair densities calculated in ref. 24 using diffusion quantum Monte Carlo, shown as DFT [TB], and the radiative lifetimes of the bright exciton, semi-dark trion and biexciton.

**Table 2:** Material parameters.

	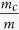 7	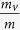 7	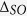 7	*A* 7	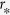 15	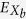 28	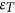 29	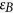 29	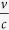 7
			[meV]	[Å^2^]	[nm]	[eV]	[meV]	[meV]	
WS_2_	0.26	−0.35	32	8.65	3.8	2	34	24	1.7 × 10^−3^
WSe_2_	0.28	−0.36	37	9.38	4.5	1.7	31	20	1.6 × 10^−3^

Listed are the effective *c*- and *v*-band electron masses *m*_*c*_ and *m*_*v*_, *c*-band spin-orbit splitting Δ_*SO*_, unit cell area *A*, 2D screening length 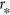, bright exciton energy 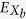, trion binding energy 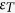, biexciton binding energy 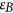, and the velocity related to the off diagonal momentum matrix element relative to the speed of light *v/c*.
