# Supplementary material for: Dark trions and biexcitons in WS2 and WSe2 made bright by e-e scattering
Source: Sci Rep. 2017 Apr 6;7:45998. doi: 10.1038/srep45998 (PMC5382684; doi:10.1038/srep45998)
Supplement: Supplementary Materials [file srep45998-s1.pdf]

Supplementary material: “Dark trions and biexcitons in $\mathrm{WS_2}$ and $\mathrm{WSe_2}$ made bright by e-e scattering”

Mark Danovich[1], Viktor Zólyomi[1] & Vladimir I. Fal'ko[1]

[1]*National Graphene Institute, University of Manchester, Booth St E, Manchester M13 9PL, UK*

S1 Group theory analysis of excitons, trions and biexcitons in Tungsten dichalcogenides

S1.1 Introduction

Group theory allows to utilize the symmetry properties of the Hamiltonian in order to gain insight into selection rules for microscopic processes in quantum systems. As a starting point, the eigenstates of the Hamiltonian are classified according to the irreducible representations (IrReps) of the symmetry group, in our case the point group C_{3h}. In monolayer TMDCs, DFT calculations[1,2] (see also S2.1) have found that band edges of monolayer $\mathrm{WS_2}$ and $\mathrm{WSe_2}$ are found at the two inequivalent corners, K and K' of the Brillouin zone. Hence, for the sake of their classification we consider the extended point group[3,4], $C''_{3v} = C_{3v} + tC_{3v} + t^2C_{3v}$, where t are translations by a lattice vector. This enables us to treat states of excitons and complexes at K, K' and zero momentum in the same fashion. The character table and product table for the IrReps of the extended point group C''_{3v} are

Table S1: C''_{3v} character table.

Character table for the irreducible representations (IrRep) of the extended point group C''_{3v}, and their correspondence to the conduction (c) and valence (v) band electrons states.

C''_{3v}	E	t, t^2	$2C_3$	$9\sigma_v$	$2tC_3$	$2t^2C_3$
A_1	1	1	1	1	1	1
A_2	1	1	1	-1	1	1
E	2	2	-1	0	-1	-1
E'_1 (c)	2	-1	-1	0	2	-1
E'_2 (v)	2	-1	2	0	-1	-1
E'_3	2	-1	-1	0	-1	2

given in Tables S1, S2, respectively. DFT calculations[1,2] (see also S2.1) have also found that at the K and K' valleys, the orbital composition of the Bloch states is dominated by the $z \to -z$ symmetric d-orbitals (d_0 for the c-band and $d_{\pm 2}$ for the v-band in the two valleys) of transition metal, allowing to classify the c and v-band Bloch states at the K and K' valleys as transforming according to the two dimensional IrReps of the extended point group, E'_1 and E'_2, respectively.

Using classification of the single electron states, we consider excitons, trions, and biexcitons. For this, we take direct products of the corresponding IrReps, and, then, apply the product rules for the IrReps of C''_{3v}, shown in Table S2. This group theory analysis enables us to identify excitonic basis states that can be mixed by the intervalley e-e scattering, leading to the class of semi-dark trions and biexcitons discussed in the main text.

Table S2: C''_{3v} **product table.**

Product table for the irreducible representations of the extended point group C''_{3v}.

C''_{3v}	A_1	A_2	E	E'_1	E'_2	E'_3
A_1	A_1	A_2	E	E'_1	E'_2	E'_3
A_2	A_2	A_1	E	E'_1	E'_2	E'_3
E	E	E	$A_1 \oplus A_2 \oplus E$	$E'_2 \oplus E'_3$	$E'_1 \oplus E'_3$	$E'_1 \oplus E'_2$
E'_1 (c)	E'_1	E'_1	$E'_2 \oplus E'_3$	$A_1 \oplus A_2 \oplus E'_1$	$E \oplus E'_3$	$E \oplus E'_2$
E'_2 (v)	E'_2	E'_2	$E'_1 \oplus E'_3$	$E \oplus E'_3$	$A_1 \oplus A_2 \oplus E'_2$	$E \oplus E'_1$
E'_3	E'_3	E'_3	$E'_1 \oplus E'_2$	$E \oplus E'_2$	$E \oplus E'_1$	$A_1 \oplus A_2 \oplus E'_3$

S1.2 Excitons

The exciton states transform according to the direct product representation of the c- and v-band states given by

$$E'_1 \otimes E'_2 = E \oplus E'_3. \quad \text{(S1)}$$

The 2D IrRep E corresponds to the intravalley excitons with both electron and hole residing in either the K or K' valleys, and the 2D IrRep E'_3 corresponds to the intervalley excitons with the electron and hole residing in opposite valleys making the exciton dark due to momentum mismatch. By further introducing the spin projections of the electron and hole, we have for each representation two possible total spin projections, $|S_z| = 1$ corresponding to dark excitons due to spin conservation, and $S_z = 0$ corresponding to bright exciton states. Using the notation intro-

duced in the text for trions and biexcitons, the E IrRep dark intravalley exciton states are given by $[X^{\uparrow K}_{\downarrow K}; X^{\downarrow K'}_{\uparrow K'}]$ with $|S_z| = 1$, and the bright intravalley excitonic states by $[X^{\uparrow K}_{\uparrow K}; X^{\downarrow K'}_{\downarrow K'}]$ with $|S_z| = 0$. Similarly, for the intervalley excitons transforming according to E'_3, which are dark due to momentum conservation, we have $[X^{\uparrow K}_{\uparrow K'}; X^{\downarrow K'}_{\downarrow K}]$ with $S_z = 0$, and $[X^{\uparrow K}_{\downarrow K'}; X^{\downarrow K'}_{\uparrow K}]$ with $S_z = 1$, being dark due to both spin and momentum conservation.

S1.3 Trions

Next we classify the trion states composed of two electrons and a hole. The strongly bound trion states require the two-electron wave function to be symmetric with respect to exchanging the electrons coordinates and the two electrons to have different spin/valley indices corresponding to a singlet state, as obtained in ref. 5 using Monte Carlo calculations. The two-electron state transforms according to the direct product of the c-band electrons representations given by

$$E'_1 \otimes E'_1 = A_1 \oplus A_2 \oplus E'_1. \tag{S2}$$

According to Table S1, the symmetric combination of the two electrons transforms according to A_1 or E'_1. The identity representation corresponds to both electrons residing in opposite valleys, while the 2D IrRep E'_1 corresponds to both electrons residing in the same valley K or K'. Next, to obtain the representation of the trion we include the hole state E'_2 and take the direct product of the two electrons and the hole. This gives in the first case

$$A_1 \otimes E'_2 = E'_2, \tag{S3}$$

corresponding to the hole residing in either the K or K' valleys and the electrons residing in opposite valleys. Including the spin projection this corresponds to the following trion states, $[T^{\uparrow K}_{\downarrow K,\uparrow K'}; T^{\downarrow K}_{\downarrow K,\uparrow K'}]$ which are the semi-dark singlet ground state trions, and $[T^{\uparrow K}_{\uparrow K,\downarrow K'}; T^{\downarrow K'}_{\uparrow K,\downarrow K'}]$ which are the excited bright trion singlet states. As the excited bright and semi-dark trion states both transform according to the same E_2' IrRep, the two states can be mixed through the electron-electron intervalley scattering introduced in the main text, which transforms as the identity representation. The bright trion triplet states with both electrons in opposite valleys also transform according to the E_2' IrRep and are given by $[T^{\uparrow K}_{\uparrow K,\uparrow K'}; T^{\downarrow K'}_{\downarrow K,\downarrow K'}]$, and the dark trion triplet states (due to spin conservation) are given by $[T^{\uparrow K}_{\downarrow K,\downarrow K'}; T^{\downarrow K'}_{\uparrow K,\uparrow K'}]$. In the second case, choosing for the two-electron representation the E_1' IrRep,

$$E_1' \otimes E_2' = E \oplus E_3'. \tag{S4}$$

Here, E corresponds to states with the two electrons and hole residing in the same valley K or K'. Requiring the electrons to have opposite spin projections gives the following bright trion states $[T^{\uparrow K}_{\uparrow K,\downarrow K}; T^{\downarrow K'}_{\uparrow K',\downarrow K'}]$. E_3' corresponds to the two electrons residing in the same valley while the hole is in the opposite valley, giving the dark trion states (due to momentum conservation) $[T^{\downarrow K'}_{\uparrow K,\downarrow K}; T^{\uparrow K}_{\uparrow K',\downarrow K}]$.

S1.4 Biexcitons

The bound biexciton states are composed of a spatially symmetric wave function for the two electrons and for the two holes. This corresponds to the IrReps $A_1 \oplus E_1'$ for the two electrons, and

$A_1 \oplus E_2'$ for the two holes. Taking the direct product of the two-electron and two-hole states gives the possible representations of the biexciton states

$$(A_1 \oplus E_1') \otimes (A_1 \oplus E_2') = A_1 \oplus E_1' \oplus E \oplus E_3' \oplus E_2'. \tag{S5}$$

The states transforming according to the IrRep E correspond to both electrons and both holes residing in the same valley, similarly the E_3' IrRep corresponds to both electrons residing in the same valley and both holes residing in the opposite valley to the electrons, and finally E_2' corresponds to both electrons residing in opposite valleys, and both holes residing in the same valley. As these three cases require one of the holes to reside in the lower spin-orbit split band in order for the biexciton to be bound, we do not consider these states. Of particular interest is the A_1 representation corresponding to both electrons and both holes residing in opposite valleys. Including the spin projections this corresponds to the following biexciton state, $B^{\uparrow K,\downarrow K'}_{\downarrow K,\uparrow K'}$ which is the semi-dark (due to momentum conservation) ground state singlet biexciton, and $B^{\uparrow K,\downarrow K'}_{\uparrow K,\downarrow K'}$ which is the excited bright state singlet biexciton. As the two states transform according to the same IrRep A_1, they can also be mixed by the electron-electron intervalley scattering process as in the trions case. The biexciton triplet states are given by $B^{\uparrow K,\downarrow K'}_{\uparrow K,\uparrow K'}$ and $B^{\uparrow K,\downarrow K'}_{\downarrow K,\downarrow K'}$ both being optically bright. The biexciton states transforming according to the E_1 IrRep are bright having both electrons in the same valley and both holes in opposite valleys, $[B^{\uparrow K,\downarrow K'}_{\uparrow K,\downarrow K}; B^{\uparrow K,\downarrow K'}_{\uparrow K',\downarrow K'}]$.

Table S3: Group theory classification.

Summary of the group theory classification of excitonic complexes, X-excitons, T-trions, and B- Biexcitons, in Tungsten dichalcogenides according to the irreducible representations of the extended point group C''_{3v}.

	IrRep	States	Bright	Dark	Exciton or complex (see Fig. 2)
X	E	$[X^{\uparrow K}_{\downarrow K}; X^{\downarrow K'}_{\uparrow K'}]$		✓	X_d
		$[X^{\uparrow K}_{\uparrow K}; X^{\downarrow K'}_{\downarrow K'}]$	✓		X_b
	E'_3	$[X^{\uparrow K}_{\uparrow K'}; X^{\downarrow K'}_{\downarrow K}]$		✓	X_d
		$[X^{\uparrow K}_{\downarrow K'}; X^{\downarrow K'}_{\uparrow K}]$		✓	
T	E'_2	$[T^{\uparrow K}_{\downarrow K,\uparrow K'}; T^{\downarrow K'}_{\downarrow K,\uparrow K'}]$ } mix		✓	T_{sd}
		$[T^{\uparrow K}_{\uparrow K,\downarrow K'}; T^{\downarrow K'}_{\uparrow K,\downarrow K'}]$ } mix	✓		T^*
		$[T^{\uparrow K}_{\uparrow K,\uparrow K'}; T^{\downarrow K'}_{\downarrow K,\downarrow K'}]$	✓		T
		$[T^{\uparrow K}_{\downarrow K,\downarrow K'}; T^{\downarrow K'}_{\uparrow K,\uparrow K'}]$		✓	–
	E	$[T^{\uparrow K}_{\uparrow K,\downarrow K}; T^{\downarrow K'}_{\uparrow K',\downarrow K'}]$	✓		T
	E'_3	$[T^{\downarrow K'}_{\uparrow K,\downarrow K}; T^{\uparrow K}_{\uparrow K',\downarrow K}]$		✓	–
B	A_1	$B^{\uparrow K,\downarrow K'}_{\downarrow K,\uparrow K'}$ } mix		✓	B_{sd}
		$B^{\uparrow K,\downarrow K'}_{\uparrow K,\downarrow K'}$ } mix	✓		B^*
		$B^{\uparrow K,\downarrow K'}_{\uparrow K,\uparrow K'}$	✓		B
		$B^{\uparrow K,\downarrow K'}_{\downarrow K,\downarrow K'}$	✓		B
	E'_1	$[B^{\uparrow K,\downarrow K'}_{\uparrow K,\downarrow K}; B^{\uparrow K,\downarrow K'}_{\uparrow K',\downarrow K'}]$	✓		B

S2 Model calculations of the intervalley scattering matrix element

S2.1 Ab initio density functional theory

In the DFT calculations the wave functions were obtained in the local density approximation, using a plane-wave basis of 600 eV cutoff energy and a k-point grid of 12×12 in the 2D Brillouin zone. We used the VASP[10] code for these calculations, which employs periodic boundary conditions in three dimensions even for 2D materials; for this reason we used a large inter-layer distance of 20 Å to mimic the limit of an isolated monolayer. The form factor was calculated by post-processing the DFT wave functions, simply taking the matrix element of the bare Coulomb interaction between the initial and final states of the scattering process. In the calculation of this matrix element we neglected spin-orbit coupling.

The form factor was calculated in reciprocal space by Fourier transforming Eq. (2) in the main text, leading to a summation on the grid of reciprocal lattice vectors. This technique is sensitive to the plane-wave cutoff energy. We have therefore tested the sensitivity of the form factor to the cutoff energy by calculating it for WS_2 with an extremely reduced cutoff of 100 eV and an increased cutoff of 900 eV. We found that reducing the cutoff reduces the form factor by 10 %, while increasing the cutoff increases the form factor by 3 %.

Convergence of the calculation was also tested for the inter-layer separation. We found that

decreasing the separation to 15 Å only changes the form factors by less than 1 %.

In Fig. S1 we show the DFT calculated band structure for WS_2 and WSe_2, showing the band edges at the K point and the spin-orbit splitting. In Tables S4 and S5 we list the DFT obtained orbital decomposition of the electron states at the K/K' points in the conduction and valence bands demonstrating the dominance of the transition metal d orbitals.

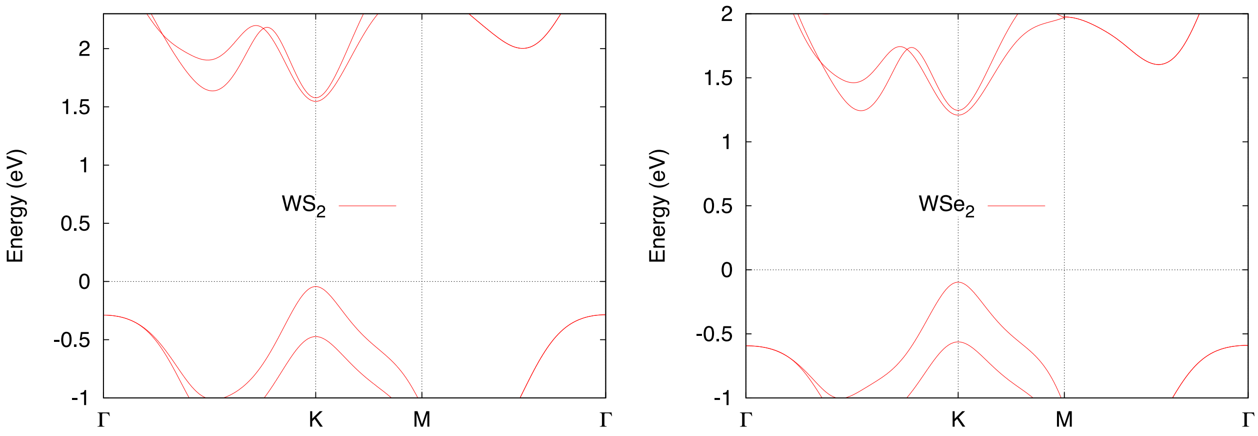

Figure S1 | DFT calculated band structure of WX_2.

Table S4 | DFT calculated orbital decomposition at the K/K' **point in** WS_2**.**

band	$\mathrm{W}-5d_{z^2}$	$\mathrm{W}-5d_{x^2-y^2}$	$\mathrm{W}-5d_{xy}$	$\mathrm{W}-6s$	$\mathrm{S}-p_x$	$\mathrm{S}-p_y$
c	86.9%	0	0	7.8%	2.6%	2.6%
v	0	39.5%	39.5%	0	10.2%	10.2%

Table S5 | DFT calculated orbital decomposition at the K/K' point in $\mathrm{WSe_2}$.

band	$\mathrm{W}-5d_{z^2}$	$\mathrm{W}-5d_{x^2-y^2}$	$\mathrm{W}-5d_{xy}$	$\mathrm{W}-6s$	$\mathrm{Se}-p_x$	$\mathrm{Se}-p_y$
c	85.9%	0	0	8.1%	2.2%	2.2%
v	0	40.1%	40.1%	0	9.2%	9.2%

S2.2 Tight-binding model

In the tight binding model, the Bloch wave function of the conduction band electrons at the K point, using only the transition metal d-orbital is given by

$$\Psi(\vec{r}) = \frac{C}{\sqrt{N}} \sum_i e^{i\vec{K}\cdot\vec{R}_i} \phi(\vec{r} - \vec{R}_i), \tag{S6}$$

where N is the number of unit cells, $\vec{R}_i$ is the lattice vector coinciding with the transition metal atoms positions, and C is the weight of the $5d_{z^2}$ orbital ϕ centred on $\vec{R}_i$. The value of C is obtained from the orbital decomposition given in Tables S4, S5 for $\mathrm{WS_2}$ and $\mathrm{WSe_2}$, respectively. The 3D coulomb matrix element is given by

$$M = e^2 \int \frac{d^3\vec{r}_1 d^3\vec{r}_2}{|\vec{r}_2 - \vec{r}_1|} \Psi^*(\vec{r}_1)\Psi^*(\vec{r}_2)\Psi(\vec{r}_1)\Psi(\vec{r}_2). \tag{S7}$$

Plugging in the Bloch wave function and using the two-centre approximation for the electron-electron Coulomb interaction we get

$$M = e^2|C|^4 \sum_{\vec{R}} e^{i\vec{K}\cdot\vec{R}} \int d^3\vec{r}_1 d^3\vec{r}_2 \frac{|\phi(\vec{r}_1)|^2|\phi(\vec{r}_2)|^2}{|\vec{r}_2 - \vec{r}_1 + \vec{R}|}, \tag{S8}$$

where the summation is over the lattice sites $\vec{R} = l\vec{a}_1 + n\vec{a}_2$, where $\vec{a}_1 = a_0(1,0)$, and $\vec{a}_2 = \frac{a_0}{2}(1,\sqrt{3})$ are the lattice primitive vectors, a_0 is the lattice constant, and l, n are integers. Finally,

the matrix element is related to the dimensionless parameter χ through the intervalley interaction Hamiltonian giving,

$$\chi = \frac{m_c}{m}\frac{A}{a_B}|C|^4 \sum_{\vec{R}} e^{i\vec{K}\cdot\vec{R}} \int d^3\vec{r}_1 d^3\vec{r}_2 \frac{|\phi(\vec{r}_1)|^2|\phi(\vec{r}_2)|^2}{|\vec{r}_2 - \vec{r}_1 + \vec{R}|}, \tag{S9}$$

where m_c is the c-band electron mass, m is the free electron mass, A is the unit cell area, and a_B is the Bohr radius.

For the atomic orbital entering into the Coulomb matrix element we use the Roothaan-Hartree-Fock (RHF) atomic orbitals[6,7] which consist of a linear combination of Slater-type orbitals,

$$\phi_{nlm}(\vec{r}) = Y_m^l(\theta,\phi)\sum_j C_j S_j(r) = Y_m^l(\theta,\phi)R_{nl}(r), \tag{S10}$$

where $n, l,$ and m are the principle, azimuthal and magnetic quantum numbers, and $Y_m^l(\theta,\phi)$ are the spherical harmonics. The Slater-type radial orbital $S(r)$ has the general form

$$S(r) = N_s r^{n-1} e^{-Zr}, \tag{S11}$$

here $N_s = \frac{(2Z)^{n+1/2}}{\sqrt{(2n)!}}$ is a normalization constant, and Z is the orbital exponent. Using the tables in ref. [7] we construct the Tungsten $5d_{z^2}$ orbital, with the radial part given by (in atomic units)

$$\begin{aligned} R_{5d}(r) = &-1070.29e^{-29.4731r}r^2 - 1297.24e^{-18.363r}r^2 \\ &+ 1192.26e^{-12.073r}r^3 + 239.385e^{-7.9781r}r^3 \\ &- 56.2785e^{-5.19312r}r^4 - 7.74766e^{-3.14551r}r^4 \\ &- 0.18956e^{-1.79159r}r^4, \end{aligned} \tag{S12}$$

and the angular part is $Y_0^2(\theta,\phi) = \frac{1}{4}\sqrt{\frac{5}{\pi}}(3\cos^2\theta - 1)$.

We separate the calculation of the matrix element into two parts, first taking $\vec{R} = 0$ giving the on-site contribution, and then allowing for $\vec{R} \neq 0$. For the on-site contribution with $\vec{R} = 0$, we expand the Coulomb potential in spherical harmonics

$$\frac{1}{|\vec{r}_2 - \vec{r}_1|} = \sum_{l=0}^{\infty} \frac{r_<^l}{r_>^{l+1}} \sum_{m=-l}^{m=l} \frac{4\pi}{2l+1} Y_m^{l\,*}(\theta',\phi') Y_m^l(\theta,\phi), \tag{S13}$$

which allows to separate the radial and angular integrations. The angular integration consists of products of three spherical harmonics which can be written in terms of Wigner 3j-symbols,

$$\begin{aligned} &\int Y_{m_1}^{l_1}(\theta,\phi) Y_{m_2}^{l_2}(\theta,\phi) Y_{m_3}^{l_3}(\theta,\phi) \sin\theta d\theta d\phi \qquad \text{(S14)} \\ &= \sqrt{\frac{(2l_1+1)(2l_2+1)(2l_3+1)}{4\pi}} \begin{pmatrix} l_1 & l_2 & l_3 \\ 0 & 0 & 0 \end{pmatrix} \begin{pmatrix} l_1 & l_2 & l_3 \\ m_1 & m_2 & m_3 \end{pmatrix}. \end{aligned}$$

The Wigner 3j-symbols impose selection rules on the possible values of the different angular momentum quantum numbers, thus reducing the number of terms in the sum and the number of integrations needed. In particular we must have, $m_1 + m_2 + m_3 = 0, |m_i| < l_i$, and $|l_1 - l_2| \leq l_3 \leq l_1 + l_2$.

For the case of non-zero $\vec{R}$, since the wave functions have a typical spread smaller than the lattice constant, we use the following expansion[8,9] valid for $|\vec{r}_1 + \vec{r}_2| < R$,

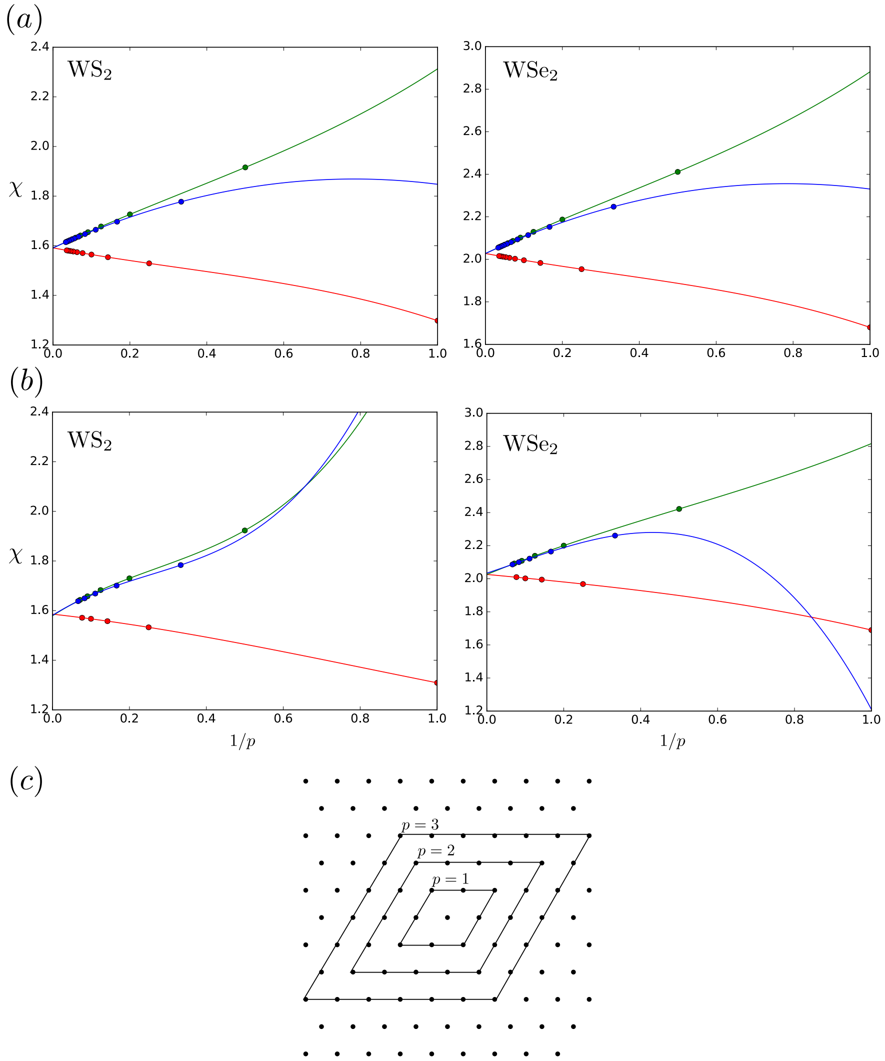

Figure S2 | Convergence of the intervalley scattering matrix element calculation.

(a) Analytical calculation of the matrix element as a function of the inverse number of lattice points in the summation. (b) Monte Carlo calculation results. We fit the points to third order polynomials and extract the value for $1/p \to 0$ corresponding to summation over an infinite lattice. The data points are separated into three sequences with a period of 3, all converging to the same point. This behaviour of the sum is attributed to the phase factor in the summation involving the $\vec{K}$ vector, and to the rhombic unit cell used in the summation. (c) Sketch of the rhombic unit cell used for the summation over the triangular lattice points for increasing values of p.

$$\frac{1}{|\vec{r}_2 - \vec{r}_1 + \vec{R}|} = \sum_{l_a,l_b=0}^{\infty} R^{-(l_a+l_b+1)} r_1^{l_a} r_2^{l_b} V_{l_a,l_b}; \tag{S15}$$

$$V_{l_a,l_b} = (4\pi)^{3/2}(-1)^{l_b} \begin{pmatrix} 2(l_a+l_b) \\ 2l_a \end{pmatrix}^{1/2}$$

$$\times [(2l_a+1)(2l_b+1)(2(l_a+l_b)+1)]^{-1/2}$$

$$\times \sum_{M=-(l_a+l_b)}^{l_a+l_b} (-1)^M Y^L_{-M}(\hat{R})[Y^{l_a}(\hat{r}_1) \otimes Y^{l_b}(\hat{r}_2)]^{l_a+l_b}_M;$$

$$[Y^{l_a}(\hat{r}_1) \otimes Y^{l_b}(\hat{r}_2)]^{l_a+l_b}_M = \sum_{m_a=-l_a}^{l_a} \sum_{m_b=-l_b}^{l_b} Y^{l_a}_{m_a}(\hat{r}_1) Y^{l_b}_{m_b}(\hat{r}_2)$$

$$\times \langle l_a m_a; l_b m_b | (l_a+l_b) M \rangle.$$

In Fig. S1 we show the convergence of the summation using both the detailed analytical method and a Monte Carlo calculation of the integral in Eq. (S9), showing that both methods converge to the same value for the dimensionless matrix element χ.

S3 Trion and biexciton oscillator strength estimation

The oscillator strength of the semi-dark trion and biexciton originates from the component of the excited bright state (T^*, B^*) in the mixed semi-dark and bright states. We express it using the oscillator strength of the exciton utilizing the fact that both the trion and biexciton can be regarded as a strongly bound exciton which is weakly bound to an electron in the trion case and another exciton in the biexciton case.

The oscillator strength is parametrized using $\alpha_{T/B}$ in Eq. (4) of the main text, giving the radiative rate of the semi-dark states in terms of the exciton radiative rate. To obtain the value of this parameter we write the excited trion and biexciton wavefunciton as a symmetrized product of an exciton and an electron in the trion case, and a symmetrized product of two excitons in the biexciton case.

For the excited trion we have,

$$\Psi_T(\mathbf{r}_{e1}^{K\uparrow}, \mathbf{r}_{e2}^{K'\downarrow}, \mathbf{r}_{h1}^{K\uparrow}) = \frac{\Psi_X(\mathbf{r}_{e1}^{K\uparrow}, \mathbf{r}_{h1}^{K\uparrow})\Psi_e(\mathbf{r}_{e2}^{K'\downarrow}) + \Psi_X(\mathbf{r}_{e2}^{K'\downarrow}, \mathbf{r}_{h1}^{K\uparrow})\Psi_e(\mathbf{r}_{e1}^{K\uparrow})}{\sqrt{2}}. \tag{S16}$$

The oscillator strength is determined to the electron-hole contact pair density, given by

$$\begin{aligned} g_{eh}^T &= \langle \Psi_T(\mathbf{r}_{e1}^{K\uparrow}, \mathbf{r}_{e2}^{K'\downarrow}, \mathbf{r}_{h1}^{K\uparrow})|\delta(r_e - r_h)\delta_{\sigma_e,\sigma_h}\delta_{\tau_e,\tau_h}|\Psi_T(\mathbf{r}_{e1}^{K\uparrow}, \mathbf{r}_{e2}^{K'\downarrow}, \mathbf{r}_{h1}^{K\uparrow})\rangle \\ &= \frac{1}{2}\int d^2\mathbf{r}_{e1}|\Psi_X(\mathbf{r}_{e1}, \mathbf{r}_{e1})|^2 = \frac{g_{eh}^X}{2}. \end{aligned} \tag{S17}$$

where σ_e, σ_h are the spins of the electron and hole, τ_e, τ_h are the valley indexes, and g_{eh}^X is the electorn-hole contact pair density of the exciton. Therefore we get $\alpha_T = 1/2$.

Similarly, for the biexciton

$$\Psi_B(\mathbf{r}_{e1}^{K\uparrow}, \mathbf{r}_{e2}^{K'\downarrow}, \mathbf{r}_{h1}^{K\uparrow}, \mathbf{r}_{h2}^{K'\downarrow}) = \frac{\Psi_X(\mathbf{r}_{e1}^{K\uparrow}, \mathbf{r}_{h1}^{K\uparrow})\Psi_X(\mathbf{r}_{e2}^{K'\downarrow}, \mathbf{r}_{h2}^{K'\downarrow}) + \Psi_X(\mathbf{r}_{e2}^{K'\downarrow}, \mathbf{r}_{h1}^{K\uparrow})\Psi_X(\mathbf{r}_{e1}^{K\uparrow}, \mathbf{r}_{h2}^{K'\downarrow})}{\sqrt{2}}. \tag{S18}$$

The corresponding electron-hole contact pair density

$$g_{eh}^B = \frac{1}{2}\left(\int d^2\mathbf{r}_{e1}|\Psi_X(\mathbf{r}_{e1}, \mathbf{r}_{e1})|^2 + \int d^2\mathbf{r}_{e2}|\Psi_X(\mathbf{r}_{e2}, \mathbf{r}_{e2})|^2\right) = g_{eh}^X. \tag{S19}$$

The two terms in the parenthesis come from the two excitons in the two vallyes both being able to recombine, giving $\alpha_B = 1$.

1. Liu, G. B., Xiao, D., Yao, Y., Xu, X. and Yao, W. Electronic structures and theoretical modelling of two-dimensional group-vib transition metal dichalcogenides. *Chem. Soc. Rev.*, 44:2643–2663, 2015.

2. Kormanyos, A. et al. k.p theory for two-dimensional transition metal dichalcogenide semiconductors. *2D Materials*, 2(2):022001, 2015.

3. Danovich, M., Zólyomi, V., Fal'ko, V. I, and Aleiner, I. L. Auger recombination of dark excitons in ws 2 and wse 2 monolayers. *2D Materials*, 3(3):035011, 2016.

4. Basko, D. M. Theory of resonant multiphonon Raman scattering in graphene. *Phys. Rev. B*, 78:125418, Sep 2008.

5. Syniszewski, M., Mostaani, E., Drummond, N. D., Aleiner, I., and Fal'ko, V. I. Binding energies of trions and biexcitons in two-dimensional semiconductors from diffusion quantum Monte Carlo calculations. *Phys. Rev. B*, 95:081301, Feb 2017.

6. Wu, Y., Tong, Q., Liu, G. B., Yu, H., and Yao, W. Spin-valley qubit in nanostructures of monolayer semiconductors: Optical control and hyperfine interaction. *Phys. Rev. B*, 93:045313, Jan 2016.

7. McLean, A. D. and R. S. McLean, R. S. Roothaan–hartree–fock atomic wave functions slater basis-set expansions for z=55–92. *Atomic Data and Nuclear Data Tables*, 26(3–4):197–381, 1981.

8. Solov'yov, I. A., Yakubovich A. V., Solov'yov, A. V. and Greiner, W. Two-center-multipole expansion method: Application to macromolecular systems. *Phys. Rev. E*, 75:051912, May 2007.

9. Paolo, A. *Intermolecular Forces and Their Evaluation by Pertubation Theory*, volume 25 of *Lecture Notes in Chemistry*. Springer, Berlin, 1981.

10. Kresse, G. and Furthmüller, J. Efficient iterative schemes for *ab initio* total-energy calculations using a plane-wave basis set. *Phys. Rev. B*, 54:11169–11186, Oct 1996.
